# Successful Management of Neutropenic Sepsis Is Key to Better Survival of Patients With Blood Cancer in Sri Lanka: Real-World Data From the Resource-Limited Setting

**DOI:** 10.1200/GO.23.00412

**Published:** 2024-03-14

**Authors:** Saman Hewamana, Thurairajah Skandarajah, Chathuri Jayasinghe, Samadhi Deshapriya, Dilip Gayashan, Natasha Peiris, Mahesh Harischandra, Prasanna Gunasena, Gnani Somasundaram, Vadivelu Srinivasan, Surjit Somiah, Chandana Wickramarathna, Sangeetha Hewawasam, Jayantha Balawardena, Gehan Arseculeratne, Rohini Wadanamby, Geethani Galagoda, Bandula Wijesiriwardana

**Affiliations:** ^1^ Lanka Hospital, Colombo, Sri Lanka; ^2^ National Cancer Institute, Colombo, Sri Lanka; ^3^ Department of Statistics, University of Sri Jayewardenepura, Colombo, Sri Lanka; ^4^ Lanka Hospital Diagnostics, Colombo, Sri Lanka; ^5^ Asiri Surgical Hospital, Colombo, Sri Lanka; ^6^ Faculty of Medicine, University of Ruhuna, Matara, Sri Lanka; ^7^ Sir John Kotelawala Defence University, Colombo, Sri Lanka

## Abstract

**PURPOSE:**

Sepsis is the main cause of nonrelapse mortality, and there are no published data on applicability of supportive care protocols from high-income countries such as Sri Lanka. The aim of the study was to investigate management and mortality of neutropenic episodes among Hemato-Oncology patients.

**MATERIALS AND METHODS:**

Retrospective analysis of clinical characteristics, management, morbidity, and mortality of neutropenic Hemato-Oncology patients presented to the Lanka Hospital Blood Cancer Centre from January 1, 2019 to December 31, 2019 was performed.

**RESULTS:**

A total of 169 neutropenic episodes were identified; 115 (68%) of such episodes were related to chemotherapy. Acute leukemia, lymphoproliferative disorders, and plasma cell disorders accounted for 23%, 69%, and 8% of patients, respectively. The median age of patients who had sepsis was 56 years, whereas that of those who had no sepsis was 53 years (*P* = .49). The median time to neutropenia was 9 days for those in the sepsis group compared with 8 days in the group that had no sepsis (0.64). The median neutrophil count in the group that had sepsis was 0.06, whereas it was 0.69 in the group that had no sepsis (*P* ≤ .05). The median time to commencement of antibiotics was 20 minutes.

**CONCLUSION:**

To our knowledge, this is the only documented study related to outcome and successful applicability of western supportive care protocols to Sri Lankan patients with neutropenia. In this study, we have shown that neutropenic sepsis can be successfully managed in the setting of limited resources with service development, following guidelines and staff training.

## INTRODUCTION

Sepsis is a common, dreaded, and costly complication in patients with cancer.^1^ According to oversimplified classification, there are three main types of blood cancers (Hemato-Oncology): leukemia, lymphoma, and myeloma, with over 100 subtypes according to 2017 WHO classification of tumors of hematopoietic and lymphoid tissues.^2^ These patients have a higher risk of acquiring infections, which have been known for decades as a main cause of death.^3^ Infections can progress rapidly with very high mortality because of impaired immune and myeloid systems. Furthermore, they are known to be at an increased risk of bloodstream infections irrespective of the neutrophil count.^4^ Because of the complexity of hematologic malignancies, complications because of treatment, and comorbidities associated with increasing age, these patients require multidisciplinary strategies and care from staff experienced in blood cancer management.

**CONTEXT**

**Key Objective**
Sepsis is a major cause of death in patients with blood cancer. Prevention and treatment of neutropenic sepsis are major challenges in the resource-limited setting. The key objective was to train staff and test the applicability of protocols from high-income countries in the resource-limited setting.
**Knowledge Generated**
To our knowledge, this is the only study on management of neutropenic sepsis in Sri Lanka. We have shown the success in the treatment of neutropenic sepsis in patients with blood cancer with training of staff and strict adherence to guidelines from high-income countries.
**Relevance**
These findings show the successful application of treatment and supportive care protocols from high-income countries in the setting of limited resources. These published data will help to benchmark and improve the treatment and develop blood cancer care in the local setting.

There is a distinct difference in the cancer survival between low- and high-income countries, and complications related to infections contribute significantly to higher morbidity and mortality in low-income countries.^5^ Sri Lanka is a developing country with a diverse health care system. Incidence of cancer has doubled over the past 25 years, and it is the second commonest cause of hospital mortality in Sri Lanka.^6^ It has a hybrid system with government-subsidized and self-financing sectors where a majority of patients seek treatment from health care workers practicing allopathic medicine and some opt to ayurvedic and other traditional ways of treatment.^7,8^ Unlike in the United Kingdom, hospitals in Sri Lanka are likely to have different approaches in managing the same disease and also significant heterogeneity exists with regard to diagnostic and treatment facilities, access to trained personnel, and supportive care. We established the Lanka Hospital Blood Cancer Centre (LHBCC) in a self-financing hospital in the capital city Colombo, Sri Lanka, in collaboration with colleagues from government-subsidized hospitals with designated space, staff, and a strategy to treat blood cancers using treatment and supportive care protocols from the United Kingdom. However, there are no published data on applicability of supportive care protocols from high-income countries in neutropenic sepsis in Hemato-Oncology patients in Sri Lanka. We used modified guidelines from high-income countries to prevent and treat neutropenic episodes in patients with blood cancer in Sri Lanka. We have previously published data on the successful application of western cancer treatment protocols in AML, Hodgkin lymphoma, and multiple myeloma.^9-11^

The aim of the present study was to investigate clinical characteristics, management, mortality, and morbidity of neutropenic episodes among Hemato-Oncology patients in LHBCC.

## MATERIALS AND METHODS

The study was considered as a quality improvement activity, and approval was obtained from the Lanka Hospitals medical research and the ethics committee. Data related to clinical characteristics, management, morbidity, and mortality of neutropenic Hemato-Oncology patients who presented to LHBCC from January 1, 2019 to December 31, 2019 were collected. Neutropenia and sepsis are previously defined in guidelines.^12,13^ The criterion of neutrophil count of 1 × 10^9^/L or lower or likely to reach below 1 × 10^9^/L because of disease or treatment was considered as neutropenia. Neutropenic sepsis was defined as patients having a temperature of 38°C persistent for 1 hour or more or other signs or symptoms consistent with clinically significant sepsis in a patient with a neutrophil count of 1 × 10^9^/L or lower. Data related to age, sex, cause for neutropenia (disease or/and chemotherapy), time of chemotherapy to neutropenia, time from neutropenia to sepsis, maximum C-reactive protein (CRP), minimum neutrophil count, minimum platelet count, minimum hemoglobin, time to antibiotics from admission or from time of sepsis (TTA), type of antibiotic, culture results, duration of antibiotic use, duration of growth factor use, duration of hospital stay, duration of neutropenia, and outcome were collected. The Mann-Whitney *U* test and the Kruskal-Wallis test were used in the statistical analysis (RStudio Team, 2020 software, Vienna, Austria); *P* values of ≤.05 were considered as the cutoff for significant difference.

Approval was obtained from the Lanka Hospitals medical and research committee.

## RESULTS

**FIG 1:**
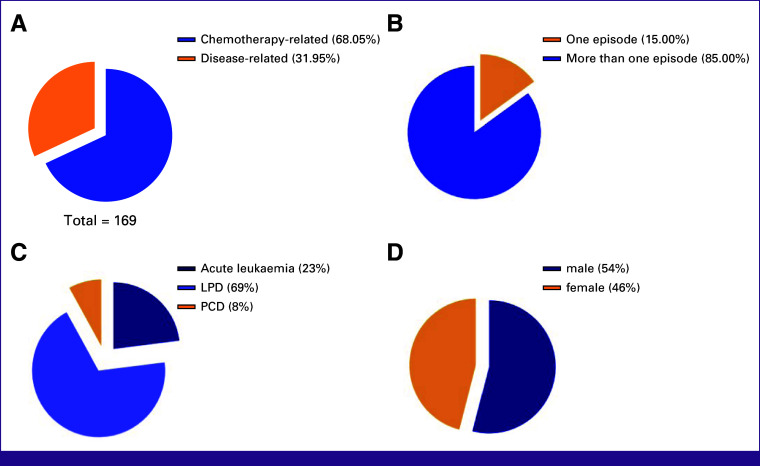
(A) Proportion of chemotherapy-related neutropenia, (B) patients who had more than one episode of neutropenia, (C) disease type, and (D) sex. LPD, lymphoproliferative disorder; PCD, plasma cell disorder.

A total of 169 neutropenic episodes were identified; 115 (68%) of such neutropenic episodes were related to chemotherapy, whereas 54 (32%) episodes were related to disease (Fig 1A). There were 43 episodes of chemotherapy-induced neutropenic sepsis (CINS), whereas 72 episodes had chemotherapy-induced neutropenia (CIN) but were not septic. In addition, there were nine episodes of disease-induced neutropenic sepsis (DINS), whereas 45 had disease-induced neutropenia but were not septic. There were 84 admissions; 52 (62%) episodes were septic, and 32 (38%) episodes admitted because of other reasons (Table 1). Fifteen percent of patients had only one episode of neutropenic sepsis during the study period, whereas 85% had more than one episode of neutropenic sepsis (Fig 1B).

**TABLE 1:** Cause of Neutropenia and Mode of Management of Neutropenic Episodes During the Study Period

Variable	Postchemotherapy, No. (%)	Disease-Related, No. (%)	Total, No.
Managed as an inpatient			
Sepsis	43 (83)	9 (17)	52
No sepsis	12 (38)	20 (62)	32
Managed in the community			
No sepsis	60 (71)	25 (29)	85
Total	115 (68)	54 (32)	169

All patients had underlying hematologic malignancy. Acute leukemia/myelodysplastic syndrome, lymphoproliferative disorders, and plasma cell disorders accounted for 23%, 69%, and 8% of patients, respectively, in the group neutropenia related to chemotherapy (Fig 1C), whereas the majority of patients were male, 54% (Fig 1D).

**FIG 2:**
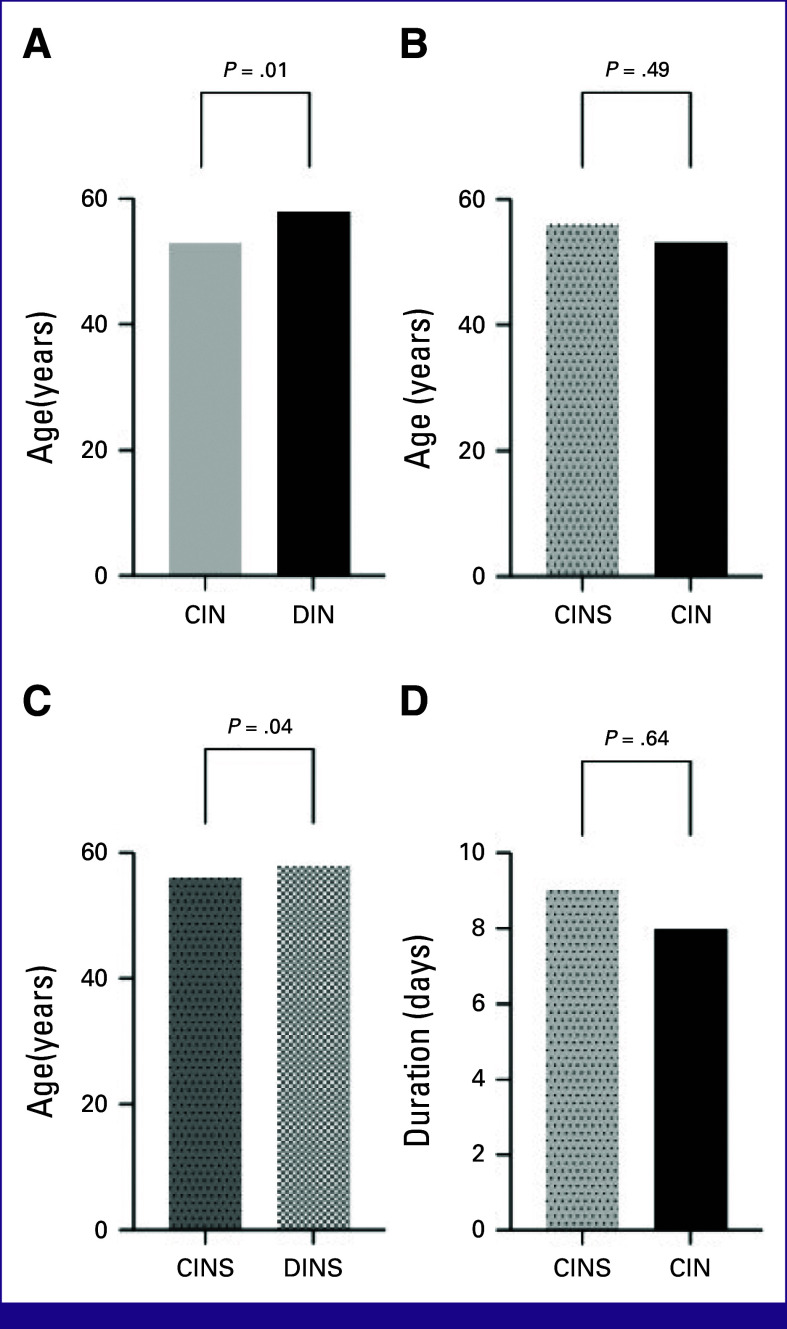
Age distribution in (A) chemotherapy-induced and disease-induced neutropenia, (B) chemotherapy-induced neutropenic sepsis and no sepsis, (C) chemotherapy-induced and disease-induced sepsis, and (D) duration in days from chemotherapy to neutropenic sepsis and neutropenia. CIN, chemotherapy-induced neutropenia; CINS, chemotherapy-induced neutropenic sepsis; DIN, disease-induced neutropenia; DINS, disease-induced neutropenic sepsis.

The median age of patients who had CINS was 56 years, whereas that of those who had no sepsis was 53 years (*P* = .49; Fig 2B). The median time to neutropenia was 9 days for those in the sepsis group compared with 8 days in the group that had no sepsis (*P* = .64; Fig 2D). The median time to sepsis from neutropenia was 1 day (Table 2).

**TABLE 2:** Age and Time From CTN, NTS, CRP, N, P, H, TTA, A, G, IP, and DN in Chemotherapy-Induced and Disease-Induced neutropenia

Variable	Chemotherapy-Induced Neutropenia		Disease-Induced Neutropenia	
	Sepsis	No Sepsis	Sepsis	No Sepsis
Age, years				
Mean (SD)	50.40 (19.25)	48.56 (19.83)	64.33 (7.57)	60.13 (7.84)
Median (IQR)	56.00 (36.00)	53.00 (37.00)	58.00 (15.00)	58.00 (0.00)
*P*	CIN *P* = .49		DIN *P* = .13	
	Sepsis *P* = .04		No sepsis *P* = .01	
CTN				
Mean (SD)	9.34 (5.15)	8.54 (3.73)	NA	NA
Median (IQR)	9.00 (5.00)	8.00 (5.00)	NA	NA
*P*	CIN *P* = .64		NA	NA
NTS				
Mean (SD)	1.46 (1.120)	NA	NA	NA
Median (IQR)	1.00 (0.00)	NA	NA	NA
*P*	NA	NA	NA	NA
CRP				
Mean (SD)	84.1 (98.5)	NA	80.0 (92.4)	NA
Median (IQR)	47.6 (81.4)	NA	44.7 (133.1)	NA
*P*	Sepsis *P* = .83			
N				
Mean (SD)	0.24 (0.32)	0.62(0.29)	0.45 (0.33)	0.50 (0.36)
Median (IQR)	0.06 (0.46)	0.69 (0.52)	0.36 (0.66)	0.50 (0.55)
*P*	CIN *P* = .000		DIN *P* = .6176	
	Sepsis *P* = .018		No sepsis *P* = .02	
P				
Mean (SD)	169.3 (134.9)	186.6 (127.6)	27.00 (26.66)	48.0 (78.9)
Median (IQR)	139.0 (117.0)	183.0 (132.0)	16.00 (17.00)	20.0 (24.0)
*P*	CIN *P* = .22		DIN *P* = .47	
	Sepsis *P* = .00		No sepsis *P* = .00	
H				
Mean (SD)	9.435 (1.81)	10.100 (2.07)	7.989 (0.85)	8.61 (1.84)
Median (IQR)	9.000 (2.30)	9.850 (2.85)	8.000 (1.40)	8.80 (1.75)
*P*	CIN *P* = .05		DIN *P* = .08	
	Sepsis *P* = .01		No sepsis *P* = .00	
TTA				
Mean (SD)	28.84 (14.91)	NA	37.78 (25.87)	NA
Median (IQR)	20.00 (20.00)	NA	20.00 (40.00)	NA
*P*	Sepsis *P* = .48			
A				
Mean (SD)	5.90 (4.30)	NA	5.22 (5.45)	NA
Median (IQR)	4.00 (3.00)	NA	3.00 (5.50)	NA
*P*	Sepsis *P* = .0787			
G				
Mean (SD)	4.00 (2.51)	NA	2.86 (3.18)	NA
Median (IQR)	3.00 (4.00)	NA	1.00 (1.00)	NA
*P*	Sepsis *P* = .04			
IP				
Mean (SD)	6.74 (5.36)	NA	6.78 (7.56)	NA
Median (IQR)	5.00 (4.00)	NA	3.00 (8.50)	NA
*P*	Sepsis *P* = .2828			
DN				
Mean (SD)	4.07 (3.04)	4.37 (5.22)	NA	NA
Median (IQR)	3.00 (4.00)	2.50 (3.75)	NA	NA
*P*	CIN *P* = .35	

Abbreviations: A, duration of antibiotics; CIN, chemotherapy-induced neutropenia; CRP, C-reactive protein; CSF, colony stimulating factors; CTN, chemotherapy to neutropenia in days; DIN, disease-induced neutropenia; DN, duration of neutropenia; G, duration of CSF; H, minimum hemoglobin; IP, inpatient stay; N, minimum neutrophil count; NA, not available; NTS, time from neutropenia to sepsis; P, minimum platelet count; SD, standard deviation; TTA, time to antibiotics from admission or from the onset of sepsis.

**FIG 3:**
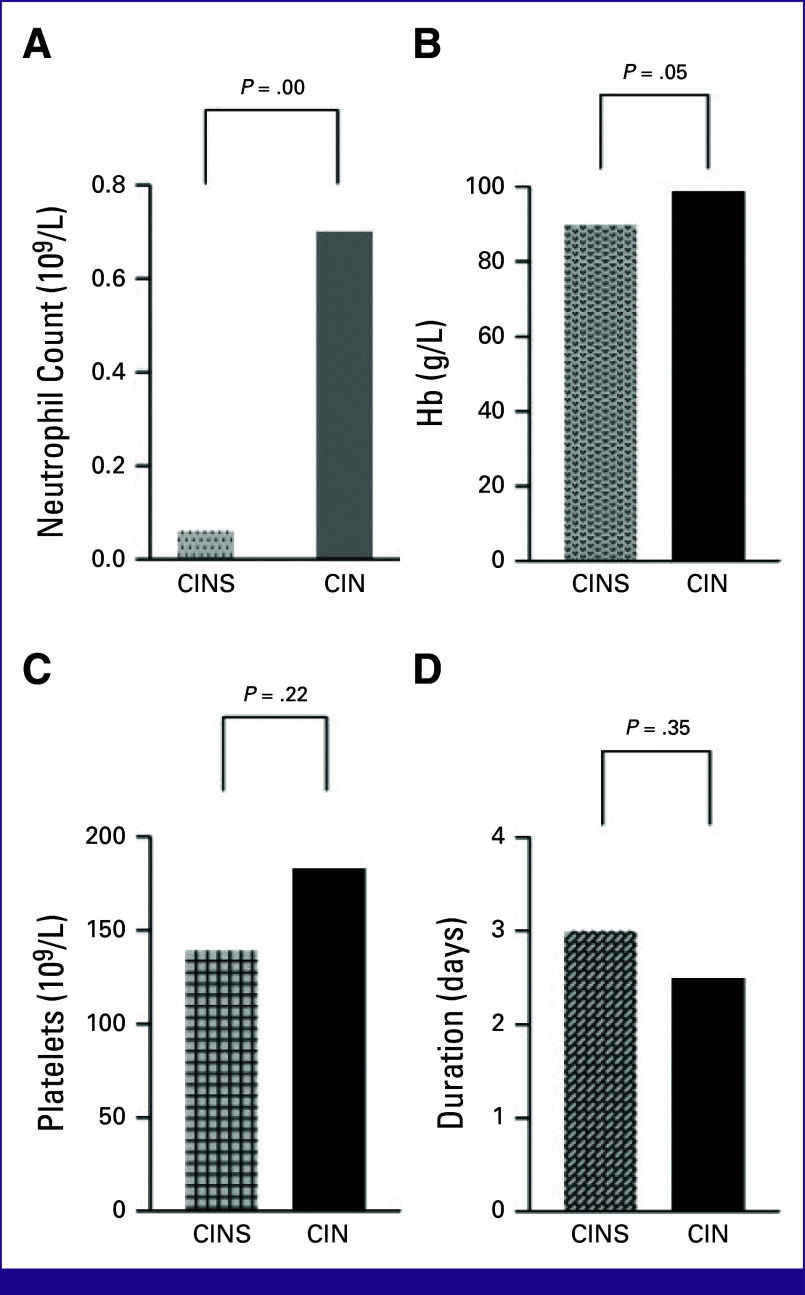
(A) Neutrophil count, (B) hemoglobin, (C) platelet count, and (D) duration of neutropenia in the chemotherapy-induced neutropenic sepsis group compared with the nonseptic group. CIN, chemotherapy-induced neutropenia; CINS, chemotherapy-induced neutropenic sepsis.

The median neutrophil count in the group that had sepsis was 0.06, whereas it was 0.69 in the group that had no sepsis (*P* < .05; Fig 3A). There was no difference between the hemoglobin, platelet count, or duration of neutropenia in the group that had sepsis compared with patients without sepsis (Table 2; Figs 3B-3D).

**FIG 4:**
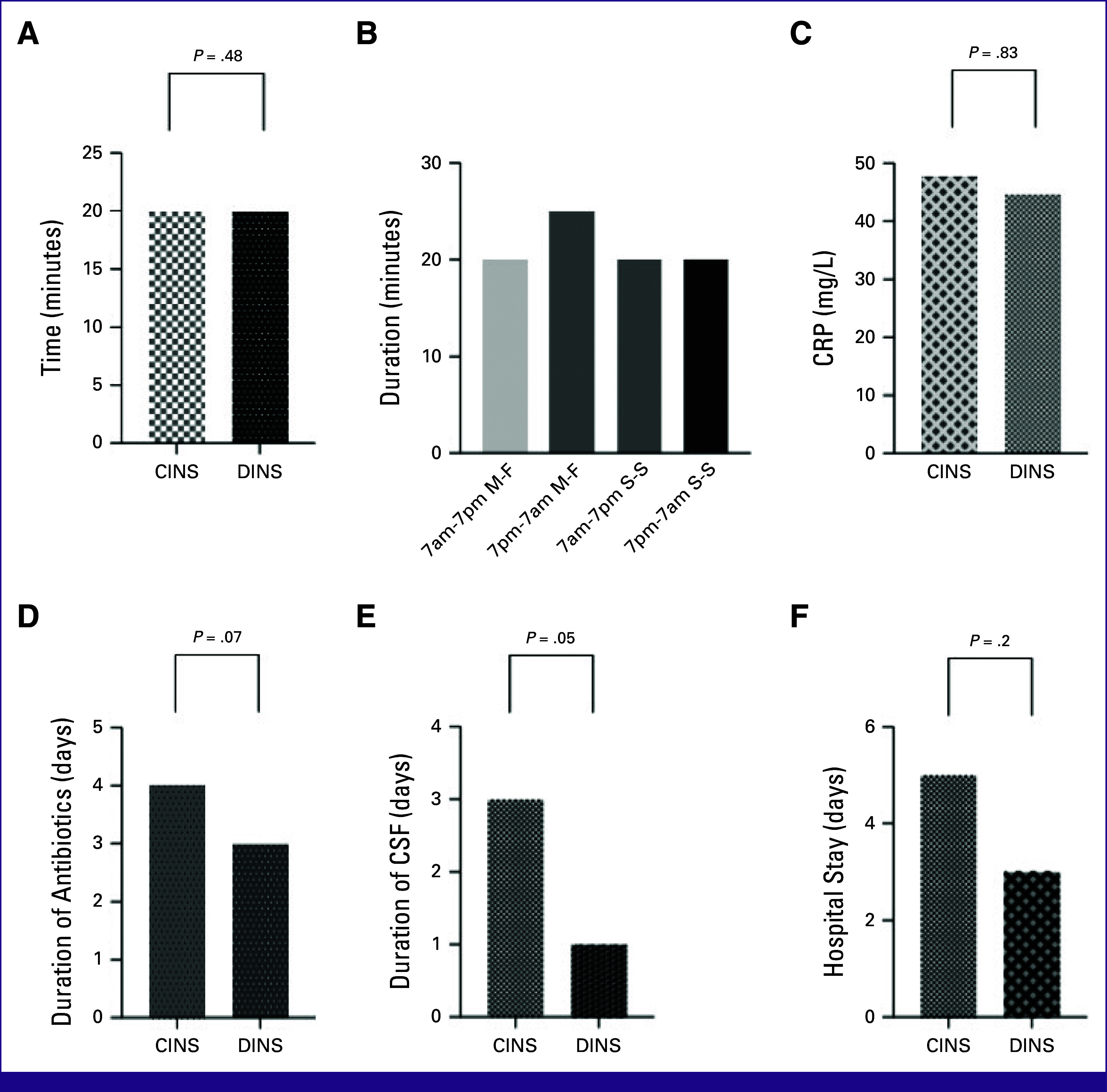
(A) TTA; (B) time to start antibiotics during daytime, night-time, and weekends; (C) CRP; (D) duration of antibiotics; (E) duration of colony-stimulating factors, and (F) duration of hospital stay in the CINS group compared with the DINS group. CINS, chemotherapy-induced neutropenic sepsis; CRP, C-reactive protein; CSF, colony stimulating factors; DINS, disease-induced neutropenic sepsis; M-F, Monday to Friday; S-S, Saturday to Sunday; TTA, time to antibiotics from admission or from time of sepsis.

The median duration of antibiotic therapy was 4 days, whereas that of granulocyte colony-stimulating factors (GCSF) therapy was 3 days. The median duration of neutropenia was 3 days, and the median hospital stay was 5 days. There was no difference in the CRP, median duration of antibiotic usage, CRP, GCSF usage, duration of neutropenia and hospital stay in the CINS group compared with the DINS group (Table 2; Fig 4). The median time to commencement of antibiotics was 20 minutes in both groups, CINS and DINS. There is no difference between the duration taken to commence antibiotics in the weekends compared with commencement on weekdays (0.48; Table 3).There were no deaths in the CINS group, but there were two deaths in the DINS group.

**TABLE 3:** TTA During the Weekdays, Night, and Weekends

Variable	7 am-7 pm M-F	7 pm-7 am M-F	7 am-7 pm S-S	7 pm-7 am S-S
Mean (SD)	28.06 (14.26)	30.45 (16.04)	30.00 (17.61)	23.33 (5.77)
Median (IQR)	20.00 (20.00)	25.00 (10.00)	20.00 (25.00)	20.00 (10.00)
*P*	Kruskal-Wallis *P* = .7870			

Abbreviations: M-F, Monday to Friday; SD, standard deviation; S-S, Saturday to Sunday; TTA, time to antibiotics from admission or from time of sepsis.

The combination of piperacillin/tazobactam (4.5 g four times per day) and amikacin (7.5 mg/kg twice a day) was the preferred first-line empirical treatment in most cases of neutropenic sepsis (40 of 43). Four were changed to carbapenem, and eight were treated with vancomycin in addition. Three episodes needed intensive care admission. A total of eight patients had positive blood (2), urine (4), sputum (1), and inter costal drain cultures in the neutropenic sepsis group (Tables 4 and 5).

**TABLE 4:** Number of Culture-Positive Episodes and Source of infection During the Study Period

Source	Neutropenic Sepsis, No.	Non-Neutropenic Sepsis, No.	Total, No.
Blood	2	2	4
Urine	4	1	5
Sputum	1	3	4
Other	1		1

**TABLE 5:** Source, Organism, and Antibiotic Sensitivity of Culture-Positive Episodes During the Study Period

Source	Organism	Antibiotic Sensitivity
Blood	*Klebsiella pneumoniae*	Amikacin, meropenem, pip + taz, vanco, teicoplanin
Blood	*Pseudomonas aeruginosa*	Amikacin, meropenem, pip + taz
Blood	*Candida species*	Amphotericin B, fluconazole, itraconazole
Blood	*Enterococcus species*	Linezolid, vancomycin, teicoplanin
	*Candida species*	Amphotericin B, fluconazole, itraconazole
Urine	*Candida species*	Fluconazole
Urine	*Klebsiella pneumoniae*	Gentamycin, pip + taz, amikacin, cotrimoxazole, ciprofloxacin
Urine	*Pseudomonas aeruginosa*	Amikacin, netilmicin, pip + taz
Urine	*Pseudomonas aeruginosa*	Gentamycin, ceftriaxone, ciprofloxacin, amikacin, pip + taz
Urine	*Klebsiella pneumoniae*	Gentamycin, cefotaxime, ciprofloxacin
Sputum	*Acinetobacter species*	Colistin
Sputum	*Candida albicans*	Fluconazole
Sputum	*Candida albicans*	Fluconazole
Sputum	*Candida species*	Fluconazole
Other (ICD)	*Staphylococcus*	Tetracycline, clindamycin, fusidic acid
	*Pseudomonas aeruginosa*	Mupirocin, gentamycin, amikacin, pip + taz

Abbreviations: ICD, intercostal drain; pip + taz, piperacillin + tazobactam.

## DISCUSSION

Developing countries lack necessary knowledge, medical practitioners, infrastructure, quality medications, and finances, which contributes to a shortage of accessible healthcare. There are no standard local protocols, guidelines, or guidelines suited for low-income countries, resulting in variability in practice and inferior patient outcome.^14^

Sepsis is a major cause of nonrelapse mortality in patients with hematologic cancers and has been shown to have three-fold higher incidence of sepsis compared with general oncology patients.^15,16^ Furthermore, the average cost of hospitalization because of neutropenic sepsis in patients with hematologic malignancies is higher than that in patients with solid tumors.^17^ It is a well-recognized complication of chemotherapy, and various measures were implemented to prevent this.^18,19^

Sri Lanka is a low-income country with a heterogeneous health care system. There are no local guidelines on prevention and treatment of neutropenia in Hemato-Oncology patients. Although there are international guidelines with recommendations, decision making in clinical practice is a challenge because of diverse population with varying comorbidities and health care facilities. Furthermore, there is scarcity of statistics and published data on response, survival, or treatment-related mortality in patients with blood cancer in Sri Lanka.

Patients receiving chemotherapy for hematologic malignancies have prolonged neutropenia, and most patients experience at least one episode of neutropenic sepsis. Neshe and Rolston^20^ showed that bacterial infections generally occur during early stage of neutropenia (7-10 days). In line with published data, 85% of patients in our cohort had more than one episode of sepsis, where median time to neutropenia from chemotherapy was 9 days and the onset of neutropenia to sepsis was only 1 day.

Weycker et al^21^ showed that the risk of febrile neutropenia during the chemotherapy regimen course was 16.8% in the United States. They analyzed solid cancers and non-Hodgkin Lymphoma (NHL). However, in line with previous publications by Caggiano et al,^17^ 36% of patient episodes with NHL in our cohort had neutropenic sepsis. The mean hospital stay in the study by Weycker et al^21^ was 8.4 days. Comparatively, hospital stay in our study was 5 days, whereas the mean duration of neutropenia was 3 days. However, direct comparison was not possible because of heterogeneity of the patient population in the two studies.

Forty one percent of episodes of neutropenia in patients with acute leukemia in our cohort had neutropenic sepsis compared with 35%-48% in AML and 13%-30% in acute lymphoblastic leukemia reported by others.^22^ However, it is not possible to differentiate the contribution by chemotherapy and disease to neutropenia, particularly during the first cycle of chemotherapy in acute leukemias.

Severe neutropenia (absolute neutrophil count of <0.1 × 10^9^/L) is associated with increased risk of severe infections.^23,24^ However, others have not seen a significant association between neutrophil count and severe infections.^25^ Three percent of episodes in this study in the group with chemotherapy-related neutropenia had a neutrophil count of <0.1 × 10^9^/L. There was a significant difference in the neutrophil count in the CINS group compared with the CIN group in our study. In addition, we could see a significant difference in hospital admission in the group with severe neutropenia.

Prevention and appropriate treatment of neutropenia and neutropenic sepsis are of great importance to maintain dose intensity and continuation of treatment according to schedules. In addition, there are medical, psychologic, and financial benefits of staying home.^26^ It has been shown that inpatient management of neutropenic complications of myelosuppressive chemotherapy costs more than chemotherapy.^27^ Prophylactic antibiotics, neutropenic diet, and growth factors are used to prevent patients’ progress to neutropenic sepsis as advised by guidelines from high-income countries.^24,28^ However, data from clinical trials may not be representative of real-world scenarios and may not be applicable to a wider population.^29^

We used ciprofloxacin prophylaxis in all patients when the neutrophil count was below 1 × 10^9^/L. We were aware that the risk of Gram-negative blood stream infections with quinolone resistance is on the rise, and this may be due to higher ciprofloxacin usage in hematology.^15^ However, we considered all patients in LHBCC as high risk because of heterogenous nature in health care facilities available, uncertainty about outcome because of lack of published data, and diverse socioeconomic status. In addition, substantial financial implications because of admissions to a self-financing hospital in Sri Lanka are also a factor considered in implementing stringent preventive measures.

Nucci et al^30^ showed that itraconazole prophylaxis reduces the frequency of systemic fungal infections and use of empirical amphotericin B in a double-blind, randomized, placebo-controlled study. We used itraconazole prophylaxis in all patients with a diagnosis of acute leukemia, and in line with their findings, we had no proven fungal infections and used amphotericin B on no patients during the study period. It is also worth noting that some newer azole antifungals like posaconazole are not readily available in Sri Lanka.

Use of GCSF is recommended when the risk of CINS is 20% or greater, but there is variability in the start time and duration of GCSF.^29^ We used GCSF in all patients with nonmyeloid malignancies with CIN. Wider use of GCSF in our center is not in line with and may be inappropriate according to studies and guidelines from high-income countries. However, we believe that appropriateness should be assessed according to cost-effectiveness, expertise, and facilities available locally. In addition, similar data are reported in the real-world practice by others^31^ and National Comprehensive Cancer Network guidelines advocate wider use of GCSF.^32^

Fresh fruit and vegetables contain Gram-negative bacilli that can cause life-threatening infections.^33,34^ However, subsequent studies have documented that neutropenic diet given in the belief that it can prevent infections has no effect.^35^ We allowed only well-cooked food in neutropenic patients in LHBCC. Once again, it is questionable how applicable studies performed in high-income countries are in the local setting where differences exist between sanitary practices including disposal of human waste and access to safe drinking water.

Gram-negative coverage in neutropenic sepsis has evolved from beta-lactam plus aminoglycoside to single-agent broad spectrums such as cefepime, piperacillin-tazobactam, and carbapenems.^36^ However, we used nurse-led first dose with dual anti–Gram-negative antibiotic treatment policy in suspected or proven neutropenic sepsis. We used piperacillin-tazobactam and aminoglycoside combination as the preferred empirical antibiotic regimen. Our policy to offer dual Gram-negative cover is mainly driven by availability of facilities. We did not have specialized trainees, but patient care was covered by generalists. Cost of treatment in intensive care is substantial in self-financed hospitals, and bed availability in the government-subsidized centers is limited. In a country with a heterogenous heath care system and sparse resources, we considered that it is safer to start dual antibiotic therapy and change over to single-agent or oral therapy once patients were stable.

Guarana et al^37^ in 2019 reported that shock or early death was mainly due to Gram-negative infections but was not associated with Gram-positive bacteremia; catheter-related, skin, or soft tissue infection; or inadequate Gram-positive coverage. Despite stringent preventive measures and empirical use of antibiotics, neutropenic sepsis carries a high mortality, which can range from 2% to 21%^38^ and is higher in adults compared with children.^39^ Ghosh et al^40^ have shown a mortality rate of 19.5% in an Indian study running over 1 year. They have used single-agent cefepime or piperacillin-tazobactam/tigecycline combination as empirical agents. Thirty seven percent of neutropenic episodes in our cohort ended up as inpatients because of sepsis. There were three deaths during the study period: two were related to disease-related neutropenic sepsis (2 of 52 episodes) and one was related to non-neutropenic sepsis (1 of 16 episodes). However, no deaths were reported because of postchemotherapy sepsis during the study period.

The effect of giving antibiotics within an hour for reducing morbidity and mortality in hematologic cancers has been previously shown. Rosa and Goldani^41^ showed that time to antibiotic administration is independently associated with 28-day mortality. They have shown that starting antibiotics within 30 minutes has a lowest mortality compared with the 31- to 60-minute group, and each increase in 1 hour has increased the 28-day mortality by 18%. We had a median TTA of 20 minutes, which was within the acceptable range compared with other studies. Ali et al^42^ published a study in 2015 on the compliance of 1 hour for TTA. They had a median TTA of 45 minutes (range ± standard deviation: 10 minutes to 6 hours ± 1 hour 10 minutes) with long delays particularly over weekends. Our data did not show a significant difference over weekends or outside standard working hours (*P* = .78). We had an admission policy where patients were admitted directly to the Hemato-Oncology ward, bypassing the busy multidisciplinary single emergency unit of the hospital. A stock of antibiotics was kept on the ward, and nurses were trained to immediately treat with empirical antibiotics for suspected or proven neutropenic sepsis even before medical review or over the phone advice.

The median antibiotic days and median hospital stay were 4 and 5 days, respectively, in CINS in LHBCC. We continued antibiotics until the neutrophil count was above one in CINS but stopped it once sepsis settles in DINS. The median duration of neutropenia was 3 days in CINS and two and a half days in the CIN group. Several studies have shown that intravenous antibiotics can be stopped before the neutrophil count recovers above one.^43,44^ Social circumstances in Sri Lanka are different from those in high-income countries, and the cost of prolonged treatment self-financing hospital is substantial. Guidelines from high-income countries do not take facilities, expertise, and epidemiology of organisms or financial impact of treatment into account. We were guided by Western guidelines but with appropriate changes taking into account facilities available locally.

Understanding the epidemiology of bacterial infections enables prevention and effective treatment.^45^ An infectious etiology is only identified in 40%-50% of neutropenic fevers, with 10%-30% having bacteremia as per published data.^20,46^ We continuously investigated incidence of infections in the LHBCC to understand the changing spectrum of organisms and their antibiotic sensitivity. We had bacteremia only in four of 89 cultures performed during the study period (4%). It has been reported that positive microbiologic detection can vary considerably depending on whether they are on antibiotic prophylaxis.^22^ As reported by Klastersky et al,^22,24^ in a trial of patients with hematologic malignancies, 17% in the group given prophylaxis compared with 31% the group with no prophylaxis had positive microbiologic detection. The incidence of culture positive episodes of sepsis among our patients is lower than that reported elsewhere. However, as authors highlighted in the study by Carvalho et al,^45^ variability in reporting of rates makes comparison difficult and there is a need for reporting standardization.

The success we encountered may reflect staff and patient awareness and commitment. This was reflected in our previous publication related to supportive care in cancer^47^ in LHBCC. Clear guidelines, improved communication, and strong leadership are necessary to prevent early deaths. Having a resident clinical hematologist experienced in managing neutropenic sepsis, system for rapid clinical evaluation, facilities for intravenous administration of antibiotics, close monitoring of patients for medical complications might have contributed to the lowering mortality.

This is a single-center study, and further studies are needed over a longer period covering more centers before reaching more definitive conclusions. However, this was the only center dedicated only to Hemato-Oncology patients to our knowledge at the time of the study. In addition, we understand the importance of continuously assessing the risks and benefits of the practice and deviations from guidelines from the Western world. Furthermore, we might have to use single-agent piperacillin tazobactam and also rotate it with cefepime to reduce emergence of resistance to aminoglycosides as reported by others.^48^

In conclusion, to our knowledge, this is the only documented study related to outcome and successful applicability of western supportive care protocols to Sri Lankan patients with neutropenia. In this study, we have shown that survival data comparable with developed countries can be achieved in the setting of limited resources with service development, following guidelines and staff training. This is a limited pilot study, and we believe that these published data will help in further development of the speciality of blood cancer care in the local setting and also improving the quality of care in other local and regional centers.

## Data Availability

Data used for the study are available from the corresponding author. The data that support the findings of this study are available on request from the corresponding author.
